# Efficacy of a proprietary formulation of fipronil/(S)-methoprene/cyphenothrin against *Ixodes scapularis* tick infestations on dogs

**DOI:** 10.1186/s13071-015-0992-1

**Published:** 2015-07-17

**Authors:** Doug Carithers, William Russell Everett, Sheila J. Gross, Ruchika Solanki

**Affiliations:** Merial, Inc., 3239 Satellite Blvd, Duluth, GA 30096 USA; BerTek, Inc., Greenbrier, AR USA; Independent Contract Statistician, Piscataway, NJ USA

**Keywords:** *Ixodes scapularis*, Deer tick, Dogs, Efficacy, Tick control

## Abstract

**Background:**

Efficacy of FRONTLINE® TRITAK® For Dogs (fipronil/(S)-methoprene/cyphenothrin, Merial, Inc., Duluth, GA) against *Ixodes scapularis* was evaluated in two separate, but concurrent laboratory studies.

**Methods:**

One day after topical treatment with placebo or active, dogs (n = 24) were infested with 50 unfed adult *Ixodes scapularis* ticks, with repeat infestations on Days 7, 14, 21 and 28. The number of live ticks was counted at 6 hours post-infestation in the first study (n = 12) and at 24 hours post-infestation in the second study (n = 12).

**Results:**

Observed efficacies in study 1 were 93-99 % at 6 hour assessments on Day 1 through Day 28 and in the second study, 98-100 % at 24 hour assessments, occurring on Day 2 through Day 29.

**Conclusions:**

A single dose of FRONTLINE® TRITAK® For Dogs (fipronil/(S)-methoprene/cyphenothrin) (0.67 ml or 1.34 ml) prevented the establishment of a new infestation following treatment, as well as the repeated weekly re-infestations with *Ixodes scapularis* ticks, for 4 weeks.

## Background

Preventing tick infestations and reducing the risk of tick-transmitted diseases are important to pet owners and veterinarians for ensuring a healthy dog. Many tick species act as vectors of bacterial and viral pathogens, often causing disease in dogs and humans. The *Ixodes scapularis* tick is an important vector for parasitic and infectious diseases such as Lyme borreliosis and anaplasmosis [1,2]. *I. scapularis* is a tick species found in the eastern and central United States [3].

Of notable concern with *I. scapularis* is its ability to transmit *Borrelia burgdorferi*, the causative agent of Lyme disease, to pets and their human companions. The *Borrelia*-spirochete can typically pass from an infected tick to the mammalian-host between 48 and 72 hours after the nymph or adult attaches [4–7]. Rapidly removing or killing ticks prior to transmission of the disease causing agents is the goal, so a sustained acaricidal activity persisting throughout the entire treatment interval would be expected to ensure better prevention of tick infestations for the pet.

This paper describes the two controlled studies performed that aimed to demonstrate the efficacy of fipronil/(S)-methoprene/cyphenothrin against *Ixodes scapularis* ticks at both 6 and 24 hours after tick infestations throughout the month.

## Methods

### Experimental design

Two studies were conducted to demonstrate the efficacy of fipronil/(S)-methoprene/cyphenothrin against *I. scapularis*. These studies were performed in the United States by an experienced, independent contract research facility and were designed in accordance with standard methods for evaluating the efficacy of parasiticides for the control of tick infestations [8]. Animals were handled in compliance with both Merial Institutional Animal Care and Use Committee (IACUC) and local IACUC approvals, and were in compliance with the Animal Welfare Act. The trial facility meets USDA-APHIS animal welfare requirements.

### Animals

The studies involved 24 purpose bred canines (12 per study), each identified by a unique numerical tattoo or implanted microchip. Beagles and mixed breed dogs, with short to medium hair length were used in these studies. Study 1 included 4 males and 8 female dogs aged 0.6-7.7 years old and weighing 6.6-13.7 kg (14.5-30.3 lbs.). Study 2 included 5 males and 7 female dogs aged 0.6-7.7 years old and weighing 6.7-11.6 kg (14.8-25.5 lbs.). Studies followed a controlled, randomized block design. All dogs were in good health and none had been treated with an ectoparasiticide product, either topically or orally, within three months prior to the study and treatment. Pre-treatment tick infestations, with removal counts were performed on Day −5 to ensure that dogs were capable of maintaining adequate tick infestations and then allocated to their assigned treatment group. Dogs were not anaesthetized or sedated prior to any of the study infestations. Dogs were housed individually. Health observations were conducted daily throughout the study, which included every hour for four hours following the treatment of the fipronil/(S)-methoprene/cyphenothrin on Day 0.

### Study design

A total of 28 dogs (12 male and 16 female) were infested with approximately 50 (25 male and 25 female) *I. scapularis* ticks on Day −5, and counted 24 hours later. The four dogs with the lowest pre-treatment tick counts were not allocated. The remaining 24 dogs (10 males and 14 females) with the highest tick counts were ranked by decreasing pre-treatment tick counts. Six replicates of 4 animals each were formed. The 4 dogs with the highest pre-treatment tick counts formed replicate 1; the next 4 highest formed replicate 2, and so on, until all dogs were allocated. Within replicates, each dog was randomly allocated to one of the four treatment groups, with two treatment groups randomly assigned to each study. On the second study, prior to Day 0, it was noticed that one dog developed a localized skin infection directly in the targeted treatment site area. That dog was removed from the study, appropriately treated, and replaced in the study by the dog with the highest remaining pre-treatment tick count. Thereafter, all the dogs remained in their assigned groups for the duration of the study. Dogs were weighed prior to Day 0 and the appropriate treatment size was selected based on the animal’s weight. On Day 0, treatment was applied according to label instructions, topically by parting the hair between the shoulder blades and applying the formulation directly to the skin. In both studies the control group, Group 1, was treated with the appropriate dose of mineral oil of either 1.0 mL or 2.0 mL. The treated group, Group 2, was treated with the weight-appropriate dose of FRONTLINE® TRITAK® For Dogs (fipronil/(S)-methoprene/cyphenothrin) of either 0.67 mL or 1.34 mL.

The *I. scapularis* ticks used in the study were unfed adult ticks, 50 % male and 50 % female, and were the OSU *Ixodes scapularis* strain from Oklahoma State University Tick Rearing Facility. Dogs were treated once on Day 0 per allocation. Dogs were each infested on Days 1, 7, 14, 21 and 28 post-treatment with 50 (25 male and 25 female) live *I. scapularis* ticks, which were placed on the lateral aspect of the dog to avoid direct contact with the product application site. At either 6 or 24 hours following each infestation, dogs in Group 1 and 2 in their respective studies were thoroughly examined by hand, removing and counting all ticks on each dog. To ensure that the hand-counting and removal was complete, dogs were combed, using a fine-toothed flea comb, for 3 minutes to ensure no ticks remained. Different flea combs were used for collection with the treated and the control group dogs.

### Data analysis

All analyses and calculations were performed using SAS Version 9.3. Statistical significance was declared at a two-sided p-value of 0.05. Both geometric and arithmetic means were determined and significance calculated.

Adult tick counts were transformed to the natural logarithm of (count + 1) to calculate geometric means. Percent efficacy for each treatment group on each day was calculated as

$$ 100\ *\ \left(\mathrm{G}\mathrm{M}\mathrm{C}\ \hbox{--} \mathrm{G}\mathrm{M}\mathrm{T}\right)\ /\mathrm{G}\mathrm{M}\mathrm{C} $$

where GMC = geometric mean of the control group and GMT = geometric mean of the treated group.

Adult tick counts were also calculated via arithmetic means. Percent efficacy for each treatment group on each day was calculated as

$$ 100*\ \left(\mathrm{M}\mathrm{C}-\mathrm{M}\mathrm{T}\right)/\mathrm{M}\mathrm{C} $$

Where MC = arithmetic mean of the control group and MT = arithmetic mean of the treated group.

The transformed data were analysed using t-tests for means with poolable variances or for means with unequal variances, as appropriate; variances were compared using the maximum-F test and Satterthwaite’s Approximation was used to determine the degrees of freedom for the unequal-variance tests. When one group had zero variance, variances were declared unequal by definition. The t-test is equivalent to one-way ANOVA when variances are poolable, and is more appropriate when variances are found to be unequal. Each treated group was compared to the corresponding control group.

## Results

All animals remained in apparent good health throughout the study. Following Day 0 treatment, no animals were removed from the study. The geometric mean counts of the live ticks in the control and treated groups of the first study ranged between 22.5 to 29.7 and 0.3 to 1.9, respectively (Table 1). The geometric mean counts of the live ticks in the control and treated groups of the second study ranged between 15.7 to 29.0 and 0.0 to 0.4, respectively (Table 2). The 6-hour efficacies observed in dogs that were infested with *I. scapularis* ticks on Days 1, 7, 14, 21 and 28 were 93.2 %, 98.9 %, 98.2 %, 95.9 % and 98.9 % (Table 1). The 24-hour efficacies observed in dogs infested with *I. scapularis* on Days 1, 7, 14, 21 and 28 and counted on Days 2, 8, 15, 22 and 29 were 98.7 %, 99.1 %, 100 %, 100 % and 99.5 % (Table 2). There was a significant difference (p < 0.01) between the treated and control dogs at both 6 and 24 hour time points through Day 29 against *I. scapularis*.

**Table 1:** Geometric tick counts and percent efficacy of fipronil/(S)-methoprene/cyphenothrin against *Ixodes scapularis* 6 hours after infestation of 50 ticks

Day of tick infestation/removal	Geometric mean tick counts (min-max) Control	Geometric mean tick counts (min-max) Treated	Efficacy
Day 1	27.7 (21–42)	1.9 (0–14)	93.2 %
Day 7	23.0 (13–32)	0.3 (0–1)	98.9 %
Day 14	22.5 (12–34)	0.4 (0–3)	98.2 %
Day 21	29.7 (22–42)	1.2 (0–5)	95.9 %
Day 28	25.7 (17–32)	0.3 (0–1)	98.9 %

**Table 2:** Geometric tick counts and percent efficacy of fipronil/(S)-methoprene/cyphenothrin against *Ixodes scapularis* 24 hours after infestation of 50 ticks

Day of tick infestation/removal	Geometric mean tick counts (min-max) Control	Geometric mean tick counts (min-max) Treated	Efficacy
Day 1/2	29.0 (18–42)	0.4 (0–6)	98.7 %
Day 7/8	28.9 (19–42)	0.3 (0–3)	99.1 %
Day 14/15	26.6 (14–43)	0.0 (0–0)	100 %
Day 21/22	15.7 (4–33)	0.0 (0–0)	100 %
Day 28/29	25.6 (16–49)	0.1 (0–1)	99.5 %

## Discussion

In both studies, a single dose of fipronil/(S)-methoprene/cyphenothrin was highly effective in controlling *I. scapularis* tick infestations. Considering the standard for tick-efficacy is a geometric mean of 90 %, fipronil/(S)-methoprene/cyphenothrin outperformed this standard demonstrating a geometric mean control level at 6 hours post-infestation of >93 % efficacy, and at 24 hours post-infestation, efficacy remained >98 % throughout the duration of the studies. Even when the data were assessed using arithmetic means, efficacies were consistently above 96 % at 24 hours post infestation at every assessment point throughout the study. At 6 hours post infestation, on Day 1, arithmetic mean efficacy was 83.5 %, but maintained at > 93 % at every other 6 hour assessment throughout the study.

The current studies evaluated the fipronil/(S)-methoprene/cyphenothrin at either 6 hours or 24 hours post-infestation throughout the 4 weeks. Though more costly to do as two separate studies, with 2 separate control groups, the investigators felt that the removal-count approach offers a more accurate and precise assessment than utilization of thumb counts, a method often used in tick studies.

There are many reports on the efficacy of current veterinary topical products against *I. scapularis*, e.g. fipronil/(S)-methoprene, amitraz and pyriproxyfen, metaflumizone plus amitraz and imidacloprid + permethrin. One study assessed the efficacy of imidacloprid + permethrin against the *I. scapularis* ticks and demonstrated a geometric mean efficacy of 96.5 % at 48 hours for 30 days [9], while another study showed a geometric mean efficacy of >95 % at 48 hours for a month with the use of indoxacarb + permethrin [10]. These results are comparable to other fipronil/(S)-methoprene formulations where efficacy assessed at 48 hours post-infestation was >90 % for a month against *I. scapularis* [11,12,9]. A single dose of fipronil/(S)-methoprene/cyphenothrin is able to reach that same level of efficacy of >90 % at just 6 and 24 hours post-infestation.

## Conclusions

The studies demonstrated the efficacy of a single dose of fipronil/(S)-methoprene/cyphenothrin against *I. scapularis*. Following the single dose, new tick infestations were rapidly cleared and the residual control against ticks was provided that continued for at least a month.

- ®FRONTLINE is a registered trademark of Merial, Inc.
- ®TRITAK is a registered trademark of Merial, Inc.

## References

[1] Chomel B (2011). Tick-borne infections in dogs—an emerging infectious threat. Vet Parasitol.

[2] Varde S, Beckley J, Schwartz I (1998). Prevalence of tick-borne pathogens in *Ixodes scapularis* in a rural New Jersey County. Emerg Infect Dis.

[3] Dennis DT, Nekomoto TS, Victor JC, Paul WS, Piesman J (1998). Reported distribution of *Ixodes scapularis* and *Ixodes pacificus* (Acari: *Ixodidae*) in the United States. J Med Entomol.

[4] Piesman J, Mather TN, Sinsky RJ, Spielman A (1987). Duration of tick attachment and *Borrelia burgdorferi* transmission. J Clin Microbiol.

[5] Des Vignes F, Piesman J, Heffernan R, Schulze TL, Stafford KC, Fish D (2001). Effect of tick removal on transmission of *Borrelia burgdorferi* and *Ehrlichia phagocytophila* by *Ixodes scapularis* nymphs. J Infect Dis.

[6] Piesman J, Dolan MC (2002). Protection against Lyme disease spirochete transmission provided by prompt removal of nymphal *Ixodes scapularis* (Acari: *Ixodidae*). J Med Entomol.

[7] Greene C, Greene C (2006). Environmental factors in infectious disease. Infectious diseases of the dog and cat.

[8] Marchiondo AA, Holdsworth PA, Fourie LJ, Rugg D, Hellmann K, Snyder DE, Dryden MW (2013). World Association for the Advancement of Veterinary Parasitology (WAAVP): guidelines for evaluating the efficacy of parasiticides for the treatment, prevention and control of flea and tick infestations on dogs and cats. Vet Parasitol.

[9] Dryden MW, Payne PA, Smith V, Hostetler J (2006). Evaluation of an imidacloprid (8.8% w/w)-permethrin (44.0% w/w) topical spot-on and a fipronil (9.8% w/w)-(S)-methoprene (8.8% w/w) topical spot-on to repel, prevent attachment, and kill adult *Ixodes scapularis* and *Amblyomma americanum* ticks on dogs. Vet Ther.

[10] Efficacy of Activyl® Tick Plus for the treatment and control of various species of tick infestations on dogs. (2012). MAH-ACT-36 http://www.merck-animal-health-usa.com/binaries/ACT-36-Tick-Plus-Efficacy_tcm96-154693.pdf (accessed 15 June, 2015).

[11] Jacobson R, McCall J, Hunter J, Alva R, Irwin J, Eschner A, Boeckh A (2004). The ability of fipronil to prevent transmission of *Borrelia burgdorferi*, the causative agent of Lyme disease to dogs. JARVM.

[12] Bonneau S, Gupta S, Cadiergues MC (2010). Comparative efficacy of two fipronil spot-on formulations against experimental tick infestations (*Ixodes ricinus*) in dogs. Parasitol Res.

